# Growth Differentiation Factor‐15 Could Predict Decreased Hemoglobin in Tuberculosis Treated With Linezolid‐Containing Regimen

**DOI:** 10.1111/crj.70195

**Published:** 2026-05-08

**Authors:** Amaylia Oehadian, Prayudi Santoso, Delita Prihatni, Andini Kartikasari, Ida Parwati, Diah Handayani, Thomas Handoyo, Noorwati Sutandiyo, Bachti Alisjahbana, Dick Menzies, Rovina Ruslami

**Affiliations:** ^1^ Hematology and Medical Oncology Division, Department of Internal Medicine Dr. Hasan Sadikin General Hospital/Faculty of Medicine, Universitas Padjadjaran Bandung Indonesia; ^2^ Pulmonology and Critical Respiratory Division, Department of Internal Medicine Dr. Hasan Sadikin General Hospital/Faculty of Medicine, Universitas Padjadjaran Bandung Indonesia; ^3^ Department of Clinical Pathology Dr. Hasan Sadikin Hospital Bandung Indonesia; ^4^ Department of Pulmonology and Respiratory Medicine Faculty of Medicine Universitas Indonesia, National Center of Respiratory Persahabatan Hospital, Universitas Indonesia Hospital Jakarta Indonesia; ^5^ Pulmonology and Critical Respiratory Division, Department of Internal Medicine Dr. Kariadi General Hospital/Faculty of Medicine, Universitas Diponegoro Semarang Indonesia; ^6^ Hematology and Medical Oncology Division Dharmais Hospital/Faculty of Medicine, Universitas Indonesia Jakarta Indonesia; ^7^ Infectious and Tropical Disease Division, Department of Internal Medicine Dr. Hasan Sadikin General Hospital/Faculty of Medicine, Universitas Padjadjaran Bandung Indonesia; ^8^ Mc Gill International TB Center Montreal Chest Institute Research Institute of the McGill University Health Centre Montreal Canada; ^9^ Pharmacology Division, Department of Biomedical Science Universitas Padjadjaran Bandung Indonesia

**Keywords:** GDF‐15, hemoglobin, linezolid, RR‐TB

## Abstract

**Background:**

Linezolid effectively treats rifampicin‐resistant tuberculosis (RR‐TB) but can cause significant hematological toxicities linked to mitochondrial dysfunction in hematopoietic stem cells. Growth differentiation factor‐15 (GDF‐15) has been identified as a potential biomarker of this dysfunction. This study aimed to determine whether baseline GDF‐15 levels can predict myelosuppression in RR‐TB patients treated with linezolid.

**Methods:**

The study included patients with RR‐TB from three referral hospitals who were treated with a linezolid‐containing regimen for at least 4 weeks. Hematological parameters and GDF‐15 levels were measured at baseline as well as at the 2nd and 4th–8th weeks of treatment.

**Results:**

Ninety‐seven subjects were included in this study. By the 2nd week of linezolid treatment, GDF‐15 levels significantly increased from a baseline of 635.58 (407.31–1583.65) to 708.96 (378.09–2408.89)pg/ml (*p* = 0.003). By weeks 4–8, 65% of patients developed myelosuppression. A correlation was found between baseline GDF‐15 levels and hemoglobin reduction at weeks 4–8 (*r* = 0.4, *p* < 0.001). Baseline GDF‐15 levels > 950 pg/mL identified patients with more than a 25% reduction in hemoglobin (AUC 0.756, 95% CI 0.659–0.838).

**Conclusion:**

Baseline GDF‐15 levels were correlated with hemoglobin changes during the 4th–8th weeks of linezolid treatment. These levels can predict myelosuppression, particularly hemoglobin changes, in patients with RR‐TB undergoing long‐term linezolid therapy.

## Graphical abstract

Baseline GDF‐15 predicts linezolid‐induced myelosuppression in rifampicin‐resistant tuberculosis. Patients with higher pretreatment GDF‐15 (> 950 pg/mL) were more likely to develop significant hemoglobin decline during therapy. Measuring GDF‐15 before treatment may help identify high‐risk patients and guide safer, personalized linezolid use.

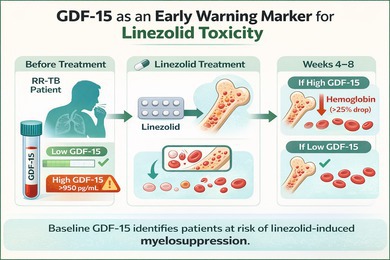

**Abbreviations**
AUC: area under the curve
BMI: body mass index
CFU‐E: colony forming unit‐erythroblast
CFU‐GM: colony forming unit‐granulocyte macrophage
CI: confidence interval
ELISA: enzyme‐linked immunoassay
GDF‐15: growth differentiation factor‐15
GP: glycoprotein
Hb: hemoglobin
IQR: interquartile range
MDR‐TB: multidrug‐resistant tuberculosis
RR‐TB: rifampicin‐resistant tuberculosis
TB: tuberculosis
WHO: World Health Organization
XDR‐TB: extensively drug‐resistant tuberculosis
βTGF‐β: transforming growth factor

## 1 Introduction

Rifampicin‐resistant tuberculosis (RR‐TB) is TB that is resistant to rifampicin detected using phenotypic or genotypic methods, with or without resistance to other anti‐TB drugs. It includes any resistance to rifampicin in the form of mono‐resistance, poly‐resistance, multidrug‐resistant tuberculosis (MDR‐TB), or extensively‐drug‐resistant tuberculosis (XDR‐TB). MDR‐TB is TB that is resistant to both rifampicin and isoniazid, the two most effective first‐line anti‐TB drugs that require a treatment regimen containing second‐line TB drugs [1]. XDR‐TB is MDR‐TB with resistance to quinolone and at least one group A TB drug i.e., linezolid or bedaquiline [2]. Linezolid is used as part of a longer regimen for MDR‐TB for 18 months [3]. In the new WHO guideline, unless contraindicated, linezolid should be the primary concern in the initial regimen for MDR/XDR‐TB [4]. Although it has good efficacy, longer treatment (more than 14 days) with linezolid causes substantial adverse events, especially hematologic toxicities [5]. Myelosuppression caused by linezolid was reported as 32.9% (95% CI 23.1%–43.5%) of MDR/XDR‐TB subjects treated with linezolid [6, 7].

Mitochondrial dysfunction plays an important role in TB infection and linezolid mechanisms of action as well as toxicities [8, 9, 10, 11]. Linezolid blocks mitochondrial ribosome protein biosynthesis and decreases adenosine thriphosphate (ATP) production in bone marrow precursor cells and further causes myelosuppression [12]. Mitochondrial dysfunction can be detected by the elevation of Growth Differentiation Factor‐15 (GDF‐15) in plasma, a divergent member of the Transforming Growth Factor‐β (TGF‐β) superfamily [13, 14].

Little is currently known about GDF‐15 role in the pathogenesis of RR‐TB. In the context of growing interest in the discovery of new biomarkers for the early detection of hematological toxicities of linezolid in RR‐TB, we aimed to explore the potential role of GDF‐15 as a predictor of hematologic toxicity in RR‐TB patients treated with linezolid to identify the possibility of this adverse event. We hypothesized that in patients with RR‐TB receiving linezolid, there would be a correlation between serum GDF‐15 levels and hematologic toxicities. Hematological parameters were measured three times during the course of treatment: baseline, the 2nd week, and the 4th–8th weeks. Plasma was collected from the patients to better understand the dynamic changes in GDF‐15 levels at baseline and the 2nd week. Because body mass index (BMI) may have an impact on hematologic parameters and GDF‐15 levels, BMI was considered for the analysis [15, 16, 17, 18, 19].

## 2 Study Design and Methods

### 2.1 Study Design

The study was a prospective cohort study conducted on 97 RR‐TB patients recruited from MDR‐TB clinics at three referral hospitals in Java (Hasan Sadikin, Kariadi, and Persahabatan hospitals), where 2/3 of MDR‐TB patients reside in Indonesia. The study was conducted between July 2021 and June 2023 and was approved by the Ethics Committee of Padjadjaran University (no. 559/UN6. KEP/EC/2021). All the participants provided written informed consent.

### 2.2 Participants and Recruitment

We included individuals with RR‐TB who were treated with a linezolid‐containing regimen for at least 4 weeks. Exclusion criteria were primary hematological disease (by history and physical examination) before diagnosis with RR‐TB and current or received myelosuppression drugs at the appropriate time of washout period. Informed consent was obtained from all individuals admitted to the clinic, screened for eligibility, and recruited consecutively.

### 2.3 Sample Size Calculation

To evaluate the correlation between GDF‐15 levels and delta hemoglobin, neutrophils, and platelets, we calculated the number of samples using a correlation analysis formula. Because there were no previous studies, we determined the minimal correlation was 0.4 with type I error of 5% and type II error of 10%. The sample size calculation was performed for 51 patients. To evaluate baseline GDF‐15 levels as a predictor of changes in hemoglobin (Hb), neutrophil, and platelet levels, the sample size was calculated using the rule of thumb for the linear regression formula (two variables: GDF‐15 levels and BMI, with Constanta = 20). The sample size resulted in 40 patients [20]. Anticipating a 10% dropout rate, we targeted a minimal sample size of 56 individuals.

### 2.4 Statistical Analysis

Numbers and percentages were used to represent categorical variables, and mean ± SD or median (interquartile range) was used to represent continuous variables when suitable. Spearman's rank correlation analysis was used to assess the relationship between changes in hemoglobin levels, neutrophil counts, platelet counts, and baseline GDF‐15 levels.

Multiple linear regression analysis was used to assess baseline GDF‐15 levels and BMI as predictors of changes in hematological parameters. To assess the variation between the baseline and second‐week GDF‐15, hemoglobin, neutrophil, and platelet counts, a paired *t*‐test or Wilcoxon test was used. We employed receiver operating characteristic (ROC) curve analysis to assess whether GDF‐15 might identify patients who will have a decrease in hemoglobin levels. Version 29.0.0.0 of IBM SPSS Statistics (version 241) was used for all statistical analyses.

### 2.5 Intervention

All eligible participants were treated with linezolid. Plasma was collected at baseline and in the 2nd week of treatment and stored at −80°C for a maximum of 3 months for GDF‐15 analysis. The plasma concentration of GDF‐15 was measured using enzyme‐linked immunosorbent assay (ELISA) at baseline and the 2nd week of treatment. Hematologic parameters were measured at baseline, at the 2nd and 4th week of treatment. Myelosuppression was defined as follows: hemoglobin < 75% of baseline, neutrophil count < 50% of baseline, and platelet count < 75% of baseline values [21].

**FIGURE 1:**
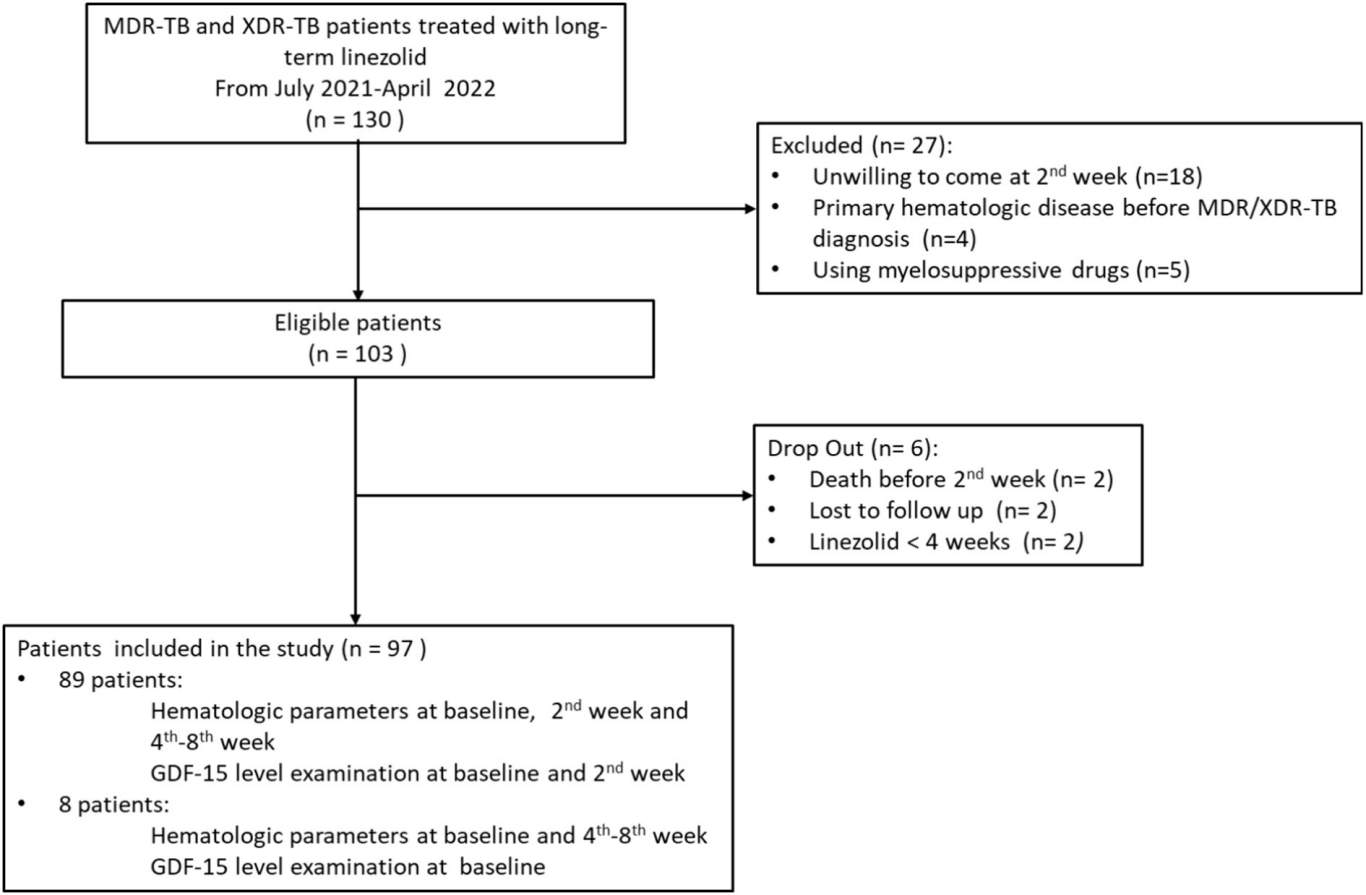
Patients flow.

Patient flow is depicted in Figure 1.

## 3 Results

From July 2021 to April 2022, there were 130 RR‐TB patients, 103 of whom were eligible for the study. Six patients were excluded for various reasons (Figure 1), leaving 97 patients included in this study. Complete hematological parameters and GDF‐15 levels were examined in 89 participants. Eight patients had only one measurement of GDF‐15 level at baseline.

Table 1 presents the baseline characteristics of the study population. The subjects were mostly male young adults, and half of them were malnourished. Smoking was found in Half of the participants smoked. More than three‐quarters of the participants were diagnosed with MDR‐TB. Type II diabetes was the most common comorbidity and was found in almost one‐fourth of the subjects. The median linezolid dose received by the subjects was 11.3 mg/kg. The baseline hematological parameters were within normal limits. Baseline GDF‐15 levels were slightly higher than normal (median 653.58 pg/mL), with a wide range (three‐fold), and almost two‐thirds of the subjects had GDF‐15 levels above normal.

**TABLE 1:** Baseline characteristics of study population.

Characteristics	Total (*n* = 103)
Age (mean ± SD)—year	39 ± 13
Male—total (*n*, %)	60 (61.9)
Body Mass Index (mean ± SD)—kg/m^2^	18.7 ± 3.3
Low body weight (BMI ≤ 18.5) *n*, %	52 (50.5)
Normal (BMI > 18.5) *n*, %	51 (49.5)
Linezolid dose (median—IQR)—mg/kg	11.3 (10.5–12.4)
Resistant type (*n*, %)	
MDR‐TB	88 (84.5)
Pre XDR‐TB	15 (15.5)
Comorbidities (*n*, %):	
Diabetes	23 (22.3)
Hypertension	8 (7.8)
Renal failure	4 (4.1)
Cardiovascular disease	3 (3.1)
Smoking (*n* = 69) (*n*, %)	
Yes	36 (52.2)
No	33 (47.8)
Hb levels (mean ± SD)—g/dl	12.8 ± 1.9
Neutrophil count (median‐IQR)—/mm^3^	7334 (2585‐14 880)
Platelets count (median‐IQR)—×1000/mm^3^	396 (149–872)
GDF‐15 levels (median‐IQR)—pg/ml (*n*, %)	653.58 (407.31–1583.65)
Normal	40 (38.8)
Above normal	63 (61.2)

Sixty‐three out of 97 (64.9%) subjects developed myelosuppression at 4–8 weeks after treatment started, mainly (65%) affecting one parameter (Table 2), mostly megakaryopoiesis disturbance (36/41, 87.8%). Erythropoiesis disturbance that led to a decrease in hemoglobin > 25% of baseline occurred in 6/63 (9.5%) patients. Three patients developed anemia.

**TABLE 2:** Myelosuppression at 4th–8th week based on hematological parameters.

Myelosuppression category (*N* = 63)	(*n*, %)
Erythropoiesis disturbance	2 (3.2)
Granulopoiesis disturbance	3 (4.8)
Megakariopoiesis disturbance	36 (57.1)
Erythropoiesis and granulopoiesis disturbances	1 (1.6)
Erythropoiesis and megakariopoiesis disturbances	2 (3.2)
Granulopoiesis and megakariopoiesis disturbances	8 (12.7)
Erythropoiesis, granulopoiesis, and megakariopoiesis disturbances	1 (1.6)

*Note:* Erythropoiesis disturbance: if hemoglobin levels < 75% baseline, granulopoiesis disturbance: if neutrophil count < 50% baseline, megakariopoiesis disturbance: if platelet count < 75% baseline.

**FIGURE 2:**
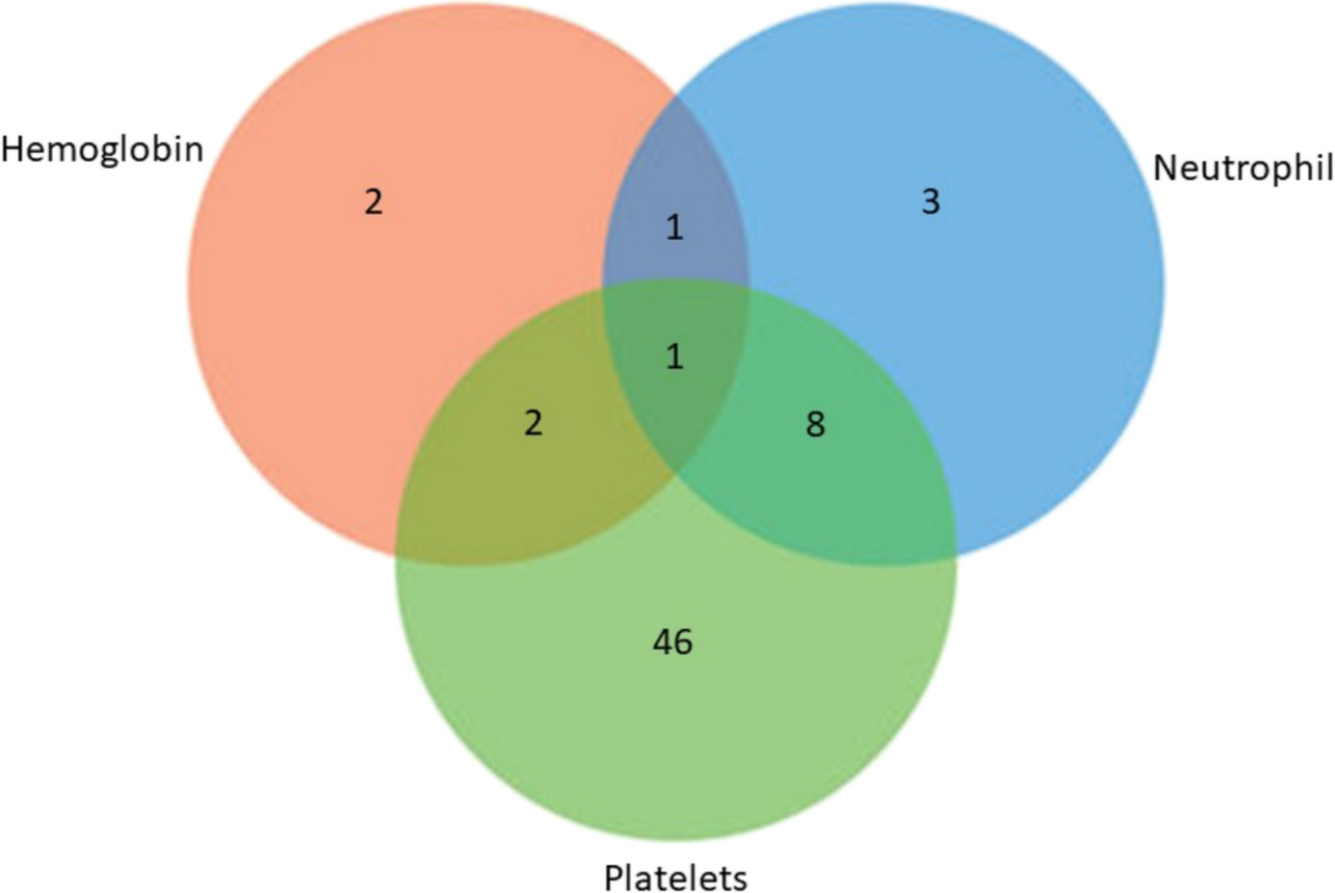
Crystal plates showed number of patients experience hematologic changes during treatment with linezolid.

Granulopoiesis disturbance that led to a decrease in neutrophil count > 25% of baseline occurred in 13/63 (20.6%) patients. Meanwhile, megakaryopoiesis disturbance that led to a decrease in platelet count > 25% of baseline occurred in 57/63 (90.4%) patients. Table 2 and Figure 2 show the distribution of subjects who developed myelosuppression in different categories. At 4th–8th weeks of treatment, the mean ± SD of hemoglobin levels was 12.0 ± 2.0 g/dL, median (IQR) of neutrophil and platelet counts were 4730 (629–15 010)/mm^3^ and 286 000 (54 000–678 000)/mm^3^, respectively.

**FIGURE 3:**
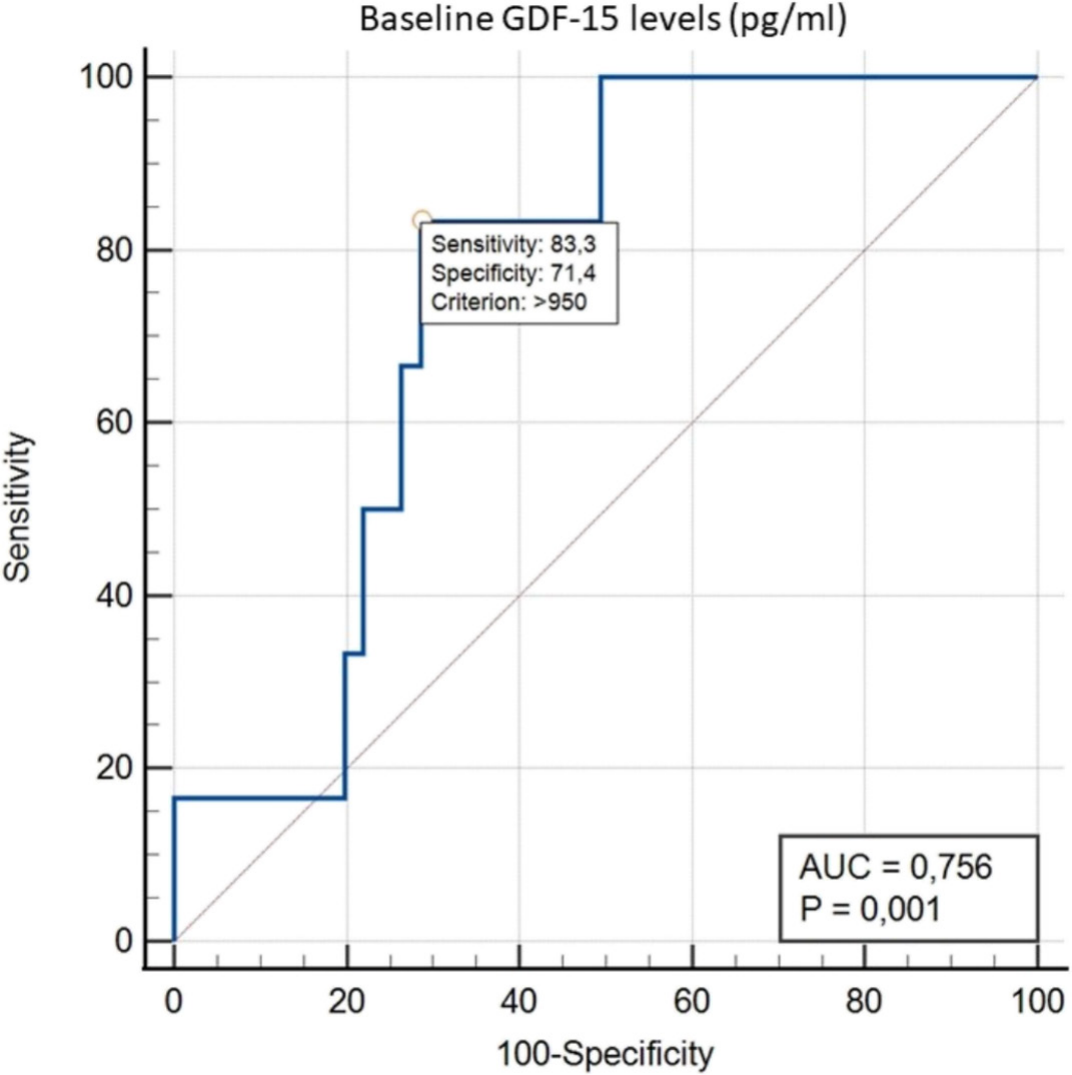
ROC curve of baseline GDF‐15 levels in discrimination patients who developed myelosuppression after linezolid treatment (hemoglobin < 75% baseline) and who did not.

Baseline GDF‐15 levels correlated moderately with hemoglobin changes at 4–8 weeks of treatment (*r* = 0.4, *p* < 0.001) and did not correlate with either neutrophil or platelet count changes (*p* > 0.05). Simple linear regression showed that baseline GDF‐15 levels could predict hemoglobin changes but not neutrophil or platelet changes. Among the subject demographics (age, sex, BMI, resistant type, and smoking status), only BMI could predict hemoglobin changes. Multiple linear regression revealed that both baseline GDF‐15 levels and BMI were independent predictors of hemoglobin changes, according to the formula: hemoglobin changes (gr/dl) = −2.310 + 0.00013 × baseline GDF‐15 levels (pg/ml) + 0.152 × BMI (kg/m^2^). By using the ROC curve, baseline GDF‐15 levels > 950 pg./mL could discriminate patients who developed myelosuppression after linezolid treatment (hemoglobin< 75% baseline) and who did not have AUC 0.756 (95% CI 0.659–0.838), 83.3% sensitivity, 71.4% specificity, 16.1% positive predictive value, and 98.5% negative predictive value (Figure 3).

**FIGURE 4:**
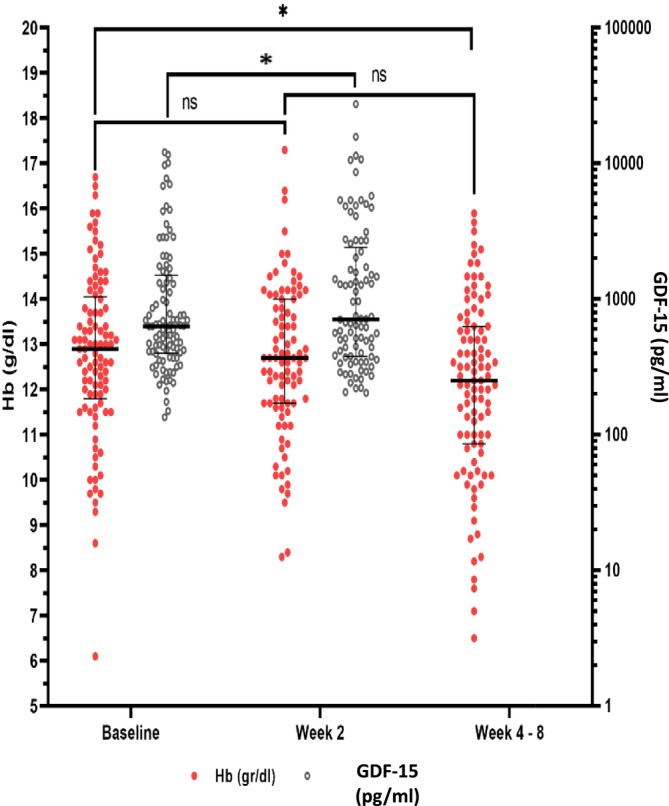
Scatter plot showing the difference between GDF15 levels and hemoglobin levels at baseline, 2 weeks and 4–8 weeks.

To understand the dynamic changes in GDF‐15 levels and hematological parameters, we measured both values at 2nd weeks of linezolid treatment. At 2nd week of treatment, GDF‐15 levels increased significantly (635.58 to 708.96 pg/mL, *p* = 0.003). On the other hand, the hemoglobin level did not change significantly (12.9 to 12.8 g/dL, *p* = 0.389) (Figure 4, Table 3).

**TABLE 3:** Changes of GDF‐15 levels and hematological parameter at the end of 2nd week.

Variable	*n*	Baseline	End of 2nd week	*p*
GDF‐15 (pg/ml)	89	635.58 (407.31–1583.65)	708.96 (378.09–2408.89)	**0.003** ^b^*
Hemoglobin levels (g/dl)	89	12.9 ± 1.8	12.8 ± 1.9	0.389^a^
Neutrophil count (/mm^3^)	89	7252 (859–9190)	5730 (4340 – 7474)	**0.001** ^b^*
Platelets count (/mm^3^)	89	384 (319–489)	279 (212–356)	**< 0.001** ^b^*

*Note:* The bold in numbers depicted statistically significant (*p* 〈 0.05).

^a^ paired *t*‐test.

^b^ Wilcoxon test.

## 4 Discussion

Our study found myelosuppression in 65% of RR‐TB patients treated with long‐term linezolid, mainly megakaryopoiesis disturbance (36/97, 37.1%). Erythropoiesis disturbance with or without other hematopoietic disturbances was found in 6/97 (6%) of RR‐TB patients treated with linezolid‐containing regimen. Baseline GDF‐15 levels were already above normal and were significantly elevated at the 2nd week of treatment. Baseline GDF‐15 levels were correlated and could predict hemoglobin changes with the formula: Hb changes (in gr/dl) = −2.310 + 0.00013 × baseline GDF‐15 levels (in pg/ml) + 0.152 × BMI (in kg/m^2^). Hemoglobin changes occurred in the 4th week of treatment, whereas GDF‐15 levels had already increased in the 2nd week. Baseline GDF‐15 levels above 950 pg/mL could identify patients who will experience hemoglobin level changes < 75% baseline with AUC of 0.756 (95% CI 0.659–0.838). These findings show the potential predictive role of GDF‐15 in hemoglobin changes in MDR/XDR TB patients treated with linezolid.

Baseline GDF‐15 levels were already higher than normal with wide inter‐individual variation [635.58 (407.31–1583.65) pg/ml], with 61% of patients having GDF‐15 levels above normal. To the best of our knowledge, this is the first report of GDF‐15 levels in patients with MDR‐TB. Growth Differentiation Factor‐15 (GDF‐15) is a member of the Transforming Growth Factor cytokine superfamily [22]. According to Tsai et al., this cytokine may be utilized as a biomarker for mitochondrial dysfunction [23]. In both TB infection and the linezolid mechanism of action, mitochondrial dysfunction plays an important role [24]. *Mycobacterium tuberculosis* (*M. tb*) causes mitochondrial disturbance in the form of size, number, form, distribution, and fragmentation [9]. Liu X, et al. reported that before treatment, GDF‐15 levels were higher in TB patients than in healthy controls [25]. Elevation of GDF‐15 levels before treatment could potentially distinguish spinal TB from mechanical back pain [26]. Linezolid inhibits bacterial protein synthesis and further interferes with the growth of bacteria by a mechanism that involves disturbance of bacterial mitochondria [27]. As expected, treatment with linezolid‐containing regimen in our patients increased GDF‐15 levels significantly at the 2nd week. Linezolid is toxic to the mitochondria of hematopoietic stem cells. Linezolid disturbs colony‐forming unit‐erythroblast (CFU‐E) and erythroblast in bone marrow, and further causes a decrease in hemoglobin [12]. Our study showed mitochondrial toxicities by elevation of GDF‐15 levels at the 2nd week after linezolid treatment. This elevation preceded a decrease in hemoglobin at the 4th–8th weeks of treatment. Erythropoiesis in the bone marrow lasts for 14 days. The development of reticulocytes into erythrocytes takes 3 days [28].

Baseline GDF‐15 levels correlated moderately with erythropoiesis disturbance, but not with other types of myelosuppression in MDR‐TB patients treated with linezolid. Apart from baseline GDF‐15 levels, changes in hemoglobin levels were also determined by BMI. Baseline GDF‐15 levels above 950 pg/mL could identify patients who will experience hemoglobin < 75% of baseline after 4th–8th weeks of treatment of linezolid with acceptable sensitivity for screening potential.

High GDF‐15 levels lead to ineffective erythropoiesis through induction of apoptosis and maturation impairment in erythroid progenitors. A high GDF‐15 level is a regulatory response mediated by proliferating erythroid cells to reduce erythroid overproduction. In the hematopoietic system, GDF‐15 expression is limited to erythroid lineage and its expression pattern alters with the erythroid maturation stage [29]. Growth differentiation factor‐15 (GDF‐15) is a cytokine upregulated in multiple pathological conditions where oxidative stress, endothelial dysfunction, tissue aging, and chronic inflammation are the hallmarks [30]. It has been shown that the high concentration of GDF‐15 is responsible for the reduced synthesis of hepcidin [31]. Hepcidin is a central regulatory peptide of iron metabolism and is associated with anemia of inflammation. Hepcidin is regulated by inflammatory cytokines, especially interleukin‐6, and its levels increase in chronic inflammation [32]. All these findings from previous studies could further explain the interplay between mitochondrial dysfunction caused by linezolid, elevated GDF‐15, hepcidin, inflammation, and hemoglobin level reduction in our study.

Growth differentiation factor 15 (GDF‐15) levels increase due to systemic inflammation and chronic disease burden. Since these biological processes are pathogenic factors of malnutrition, higher serum GDF‐15 concentrations are associated with worsening nutritional status [33]. GDF‐15 is also involved in the regulation of appetite and metabolism, which may help to explain the anorexia and weight loss [34]. Half of our patients were malnourished and these findings could contribute to elevated baseline GDF‐15 levels.

In this study, GDF‐15 levels did not correlate with granulopoiesis or megakaryopoiesis disturbances. This could be explained by another mechanism of drug‐induced myelosuppression besides disturbance of colony‐forming unit granulocyte‐macrophage (CFU‐GM) in the bone marrow. Drug‐induced neutropenia could be caused by an immune mechanism (drug‐hapten antibody) and disturbance of bone marrow stromal cells [35, 36, 37]. Besides disturbance of burst‐forming unit megakaryocytes in the bone marrow, linezolid causes thrombocytopenia by increasing phosphorylation of myocyte light chain 2 and further inhibiting the release of platelets from megakaryocytes [38, 39, 40]. Immune mechanisms that are the same as quinine/quinidine‐induced thrombocytopenia also play a role in linezolid‐induced thrombocytopenia. There is an antibody directed to glycoprotein (GP) Ib/IX and GP IIb/IIIa on the platelet membrane, which further causes increased platelet destruction [39].

We found differences in the prevalence of myelosuppression compared with previous studies. These differences could be explained by various definitions of myelosuppression, genetic factors, disease variation (TB vs. non‐TB) of study populations, and the time length of observation [21, 41, 42, 43, 44]. Erythropoiesis disturbance in our study was found in 6% (6/97) patients treated with linezolid. In our six patients who experienced erythropoiesis disturbance, linezolid was discontinued in two, and Hb levels improved at the 4th week after linezolid had been stopped. These two patients also received red cell transfusion. Linezolid was reintroduced at half dose (300 mg), and none of the three patients experienced anemia again [45]. One‐third of our patients experienced megakaryopoiesis disturbance; however, these changes were not clinically significant due to the large number of platelets.

One of the main strengths of this study is that it was conducted in three referral hospitals, reflecting several ethnicities. There are no previous studies on GDF‐15 in MDR‐TB and pre‐XDR‐TB patients. The results of this study should be interpreted in the context of several limitations. We acknowledge several study limitations, including the small size of the cohort, to evaluate other factors that could influence GDF‐15 levels, such as age, sex, diabetes, hypertension, and possible diurnal variation. We also did not examine other possible factors in the bone marrow environment that could influence hematologic changes during linezolid treatment. For further studies, it would be interesting to validate the formula before expanding its use in clinical practice to calculate Hb changes based on GDF‐15 levels and BMI.

## 5 Conclusion

GDF‐15 levels measured at baseline could possibly be used to predict Hb level changes during linezolid treatment in MDR/pre‐XDR‐TB patients. Within the 4th–8th weeks after linezolid treatment, we observed a significant decrease in Hb levels, neutrophil, and platelet counts, while GDF‐15 levels significantly increased at the 2nd week. Baseline GDF‐15 levels > 950 pg/mL could identify patients who will experience Hb level changes < 75% of baseline.

## Author Contributions

**Amaylia Oehadian:** conceptualization, data curation, formal analysis, investigation, methodology, supervision, validation, visualization, writing – original draft, writing – review and editing. **Prayudi Santoso:** conceptualization, formal analysis, investigation, methodology, supervision, validation, writing – review and editing. **Delita Prihatni:** writing – review and editing. **Andini Kartikasari:** writing – review and editing. **Ida Parwati:** writing – review and editing. **Diah Handayani:** data curation, investigation, writing – review and editing. **Thomas Handoyo:** data curation, investigation, writing – review and editing. **Noorwati Sutandiyo:** writing – review and editing. **Bachti Alisjahbana:** formal analysis, methodology, writing – review and editing. **Dick Menzies:** conceptualization, formal analysis, methodology, writing – review and editing. **Rovina Ruslami:** conceptualization, data curation, formal analysis, investigation, methodology, supervision, validation, visualization, writing – original draft, writing – review and editing. Approval of final manuscript: all authors.

## Funding

This work was supported by the Doctoral Dissertation Research Program, an internal Universitas Padjadjaran Research Funding under Grant 1595/UN6.3.1/PT.00/2021; the ALG (Academic Leadership Program), an internal Universitas Padjadjaran Research Funding under Grant 3855/UN.C/LT/2019; and the Indonesian Endowment Fund for Education (LPDP), under the EQUITY Program (Contract Nos. 4303/B3/DT.03.08/2025 and 3927/UN6.RKT/HK.07.00/2025).

## Ethics Statement

This study was approved by the Research Ethics Committee of Universitas Padjadjaran (No. 559/UN6. KEP/EC/2021). All procedures were conducted in accordance with the ethical standards of the institutional research committee, as well as the Declaration of Helsinki.

## Consent

Written informed consent was obtained from all the participants.

## Conflicts of Interest

The authors declare no conflicts of interest.

## Data Availability

Data will be made available on reasonable request. With permission from the corresponding authors, we can provide participant data without names and identifiers. The corresponding authors have the right to decide whether to share data based on the research objectives and plans provided.
